# Sucrose feeding in mouse pregnancy leads to hypertension, and sex-linked obesity and insulin resistance in female offspring

**DOI:** 10.3389/fphys.2013.00014

**Published:** 2013-02-18

**Authors:** Anne-Maj Samuelsson, Phillippa A. Matthews, Eugene Jansen, Paul D. Taylor, Lucilla Poston

**Affiliations:** ^1^Division of Women's Health, Women's Health Academic Center, King's College London and King's Health PartnersLondon, UK; ^2^Laboratory for Health Protection Research, National Institute for Public Health and the EnvironmentBilthoven, Netherlands

**Keywords:** obesity, hypertension, sucrose feeding, leptin, insulin

## Abstract

Eating an unbalanced diet during pregnancy may induce long-term health consequences in offspring, in particular obesity, insulin resistance, and hypertension. We tested the hypothesis that a maternal diet rich in simple sugars predispose mouse offspring to obesity, glucose intolerance, and cardiovascular diseases in adulthood. Female C57BL/6J mice were fed either a standard chow or a sucrose-rich diet (26% of total energy) 6 weeks prior to mating, throughout pregnancy and lactation. Offspring of control dams (OC) and high sucrose fed dams (OSF) were weaned onto standard control chow, and metabolic and cardiovascular parameters determined at 3 months of age. Both male and female OSF were hyperphagic by 4 weeks of age and females were heavier than OC at 6 weeks. At 3 months, female OSF showed a significant increase in inguinal fat pad mass, whereas skeletal muscle mass (*tibialis anterior*) and locomotor activity were decreased relative to OC. A 10-fold increase in fasting serum insulin in female OSF vs. OC at 3 months (Insulin [pmol/L] mean ± SEM, OSF, 200.3 ± 16.1, vs. OC, 20.3 ± 1.8, *n* = 6 *P* < 0.001), was associated with impaired glucose tolerance (AUC [mmol/L min] mean ± SEM, OSF 1437.4 ± 124.2 vs. OC, 1076.8 ± 83.9, *n* = 6, *P* < 0.05). Both male and female OSF were hypertensive as assessed by radiotelemetry (night-time systolic arterial pressure (SAP) [mmHg] mean ± SEM, male OSF, 128 ± 1 vs. OC, 109 ± 1, *n* = 6, *P* < 0.01; female OSF, 130 ± 1 vs. OC, 118 ± 1, *n* = 6, *P* < 0.05). Analysis of heart rate variability (HRV) demonstrated an increased low:high frequency ratio in male and female OSF (*P* < 0.05), indicative of heightened sympathetic efferent tone. Renal tissue noradrenaline (NA) content was markedly raised in the OSF vs. OC (NA [pg/ml/mg tissue] mean ± SEM, male OSF, 2.28 ± 0.19 vs. OC 0.84 ± 0.09, *n* = 6, *P* < 0.01). Exposure to a maternal diet rich in sucrose led to obesity and glucose intolerance in female mice offspring, and hypertension in both sexes.

## Introduction

The fetal “overnutrition” hypothesis suggests that an over-rich nutritional environment during the earliest stages of life may have persistent consequences for the longer-term health of the offspring (Catalano and Ehrenberg, 2006; Poston, 2012). Studies in pregnant women and their children have suggested that exposure to maternal diabetes and/or obesity increases the risk of development of insulin resistance and metabolic syndrome in the offspring (Pettitt et al., 1983; Catalano et al., 2003, 2009; Poston, 2010). Babies born to diabetic women are often macrosomic (Ehrenberg et al., 2004; Metzger et al., 2008; Segregur et al., 2009) and fatter infants may be more prone to develop obesity in childhood (Boney et al., 2005; Pirkola et al., 2010; Sparano et al., 2012).

The importance of maternal glucose control in determining fetal overweight has been suggested in the HAPO study of more than 23,000 pregnant women, in which a linear relationship between the maternal plasma glucose concentration and macrosomia was observed, even amongst non-diabetic women (Metzger et al., 2008; HAPO Study Cooperative Research Group, 2009). Infant adiposity as directly measured by the sum of skin-fold thickness, exhibited a similar strong linear relationship with maternal glucose (HAPO Study Cooperative Research Group, 2009).

Whilst obesity contributes to gestational diabetes, diet itself is likely to play an important role in the perturbation of maternal glucose homeostasis. Several studies have suggested that diets high in simple sugars exert a high glycemic load (GI), and are a major cause of obesity and metabolic syndrome (Brand-Miller et al., 2002; Schulze et al., 2004). Simple sugars e.g., sucrose and fructose such as those abundant in many soft drinks are consumed in high quantities among adolescence and adults (Nikpartow et al., 2012a,b; Wang Jensen et al., 2012). It has been reported that soft drink consumption is associated with increased prevalence of obesity, diabetes, (Moreno and Rodriguez, 2007; Nikpartow et al., 2012a) and cardiometabolic diseases (Dhingra et al., 2007; Malik et al., 2010; De Koning et al., 2012). In pregnant women, an increased dietary GI is associated with increased risk of maternal obesity and gestational diabetes mellitus (GDM) (Zhang et al., 2006). Low-glycemic diets during pregnancy may improve maternal glucose homeostasis and gestational weight gain (Walsh et al., 2012).

Surprisingly, considering the current increased prevalence of obesity and the growing interest in the role of a high GI diet in obesity (Jeppesen et al., 1997; Liu et al., 2000), the effect of exposure to a high sucrose diet *in utero* on metabolic and cardiovascular complications in the offspring has not been extensively investigated.

A study from our laboratory showed that adult offspring of obese mice developed hyperphagia, increased fat mass, hypertension, and insulin resistance (Samuelsson et al., 2008). Maternal obesity was induced by a highly palatable diet rich in sugars and animal fat. In the present study we have investigated in isolation the effects of the high sugar component of the obesogenic diet by employing a maternal diet rich in sugar but low in fat, and studied the potential adverse affects on cardiovascular and metabolic function in the offspring. We report analysis of the serum profile of a range of relevant biomarkers of metabolic and cardiovascular risk both in the dams, and in the young adult offspring.

## Methods

### Animals

All procedures involving the use of animals comply with the regulations of the United Kingdom Animals (Scientific Procedures) Act 1987, and the local animal ethics committee. Female C57BL/6J mice (Charles River Laboratories, UK, *n* = 24), proven breeders (one previous litter), were maintained under controlled conditions (20°C and 60% humidity; light dark cycle 12 h) with *ad libitum* access to food and water. After 1 week of acclimatization, female mice were fed either standard chow (7% simple sugars, 3% fat, 50% polysaccharide, 15% protein (w/w) RM1, Special Dietary Services, UK, energy 3.3 kcal/g) or standard chow supplemented with *ad libitum* access to sweetened condensed milk (55% simple sugars, 10% fat, 9% protein (w/w) 3.5 kcal/g, Nestle®, SZ) fortified with added micronutrient mineral mix (AIN93G, Special Dietary Services, UK) to achieve the same content as standard chow. Macronutrient and calorific intake were calculated from measured daily intake of the diet (approx. 5% fat, 26% simple sugars, 12% protein, energy 3.4 kcal/g). Animals were maintained on the experimental or control diet for 6 weeks before conception and throughout pregnancy and suckling. Maternal weight and dietary intake were recorded daily. At 48 h post-partum, litters were reduced to 3 male and 3 female pups to standardize the dam's milk supply during suckling. All offspring were weaned at 21 days of age onto standard chow and body weight and food intake recorded weekly. At weaning, one male and one female from each litter were fasted and humanely killed and blood samples were collected. Heart, fat pad (*inguinal*), liver, and skeletal muscle (*tibialis anterior*) weights were recorded. Metabolic and cardiovascular parameters were determined at 3 months in one male and one female per litter. The weight of organs was determined in the same animals at necropsy. Dams were fasted and humanely killed at weaning, and blood samples collected. Serum was stored at −80°C for future analysis, and the weight of organs was recorded.

### Milk leptin measurements

At weaning, a subgroup of dams, oxytocin was administered by injection (4 IU, i.p., *n* = 3 per group) to stimulate milk production. Samples of milk were obtained under anaesthesia (isofluorane) without recovery. Milk samples were stored at −20°C prior to leptin assay (see below).

### Hemodynamic measurements

Arterial pressure and heart rate were assessed at 3 months of age by remote radiotelemetry in conscious freely moving mice. Implantation of a radiotelemetry probe catheter (TA11PA-C10, O.D 0.4 mm, Data Science International Inc., St Paul, MN, US) into the aortic arch via the left carotid artery was performed under general anaesthesia (medetomidine; Domitor, Orion Espoo, Finland, 0.5 mg/kg, i.p. and ketamine; 75 mg/kg, i.p.). Pre- and post-operative analgesia (Buprenorphine, 0.1 mg/kg) was maintained for 24 h. Heart rate, systolic, and diastolic arterial pressure, and activity levels were recorded for a period of two days, after one-week recovery, by scheduled sampling for 10 s every 5 min (Dataquest LabPRO Acquisition System version 3.01, Data Sciences International). Data is reported as hourly averages for a single 24-h period. Heart rate variability (HRV) from BP signals (HR derived from pressure waves) was determined by spectral analysis. Briefly, analysis of time and frequency domains employing the HR variability Model for Chart Software (AD Instruments, Colorado Springs, CO, US) and power spectra were divided into three frequency ranges: very low frequency (VLF) zone <0.25 Hz; low frequency (LF) zone, 0.25–1.0 Hz; high frequency (HF) zone, 1.0–5.0 Hz). Integrals of the amplitude spectrum were calculated and they were used to define the spectral powers of the different frequency bands. The influence of alteration in total power of each component was prevented by normalizing the spectral power of the LF and HF zone. Normalized units were calculated by the following method: LF_nu_ = LF/(total power − VLF) × 100 and HF_nu_ = HF/(total power − VLF) × 100. The ratio of spectral powers (LF/HF) was also calculated.

### Glucose tolerance test

At 3 months of age, glucose tolerance was assessed after a single bolus of glucose (1 g/kg; i.p.) after an overnight fast. The glucose concentration of samples of blood from the tail vein was measured at 0, 15, 30, 60, 90, and 120 min in conscious, semi restrained animals (GlucoMen Sensor, A. Menarini Diagnostics, IT).

### Renal noradrenaline analysis

Animals were humanely killed at 3 months of age. The left kidney was immediately removed and rapidly frozen in liquid nitrogen. Whole kidneys were then homogenized in 0.01 M HCl in the presence of EDTA (1 mM) and sodium metabisulfite (4 mM). After centrifugation (8000 g, 30 min), a 50 μl aliquot of the supernatant was taken for noradrenaline (NA) assay by ELISA (ALPCO Diagnostics, SA, US: nr. 17-NORHU-E01-RES). NA content was expressed as pg/ml/mg renal tissue weight.

### Biochemical determinations

Samples from dams and their 3 month old offspring were analysed by autoanalyser (LX20, Beckman Coulter, Mijdrecht, Netherlands; with Beckman Coulter kits: Glucose; UV-hexokinase; nr. GLU 1442640; Triglycerides; enzymatic GPO method; nr. TG 445850; Total cholesterol; enzymatic method; nr. CHOL 467825). Samples from dams and their 3 month old offspring were assayed by insulin ELISA (Mercodia, Uppsala, Sweden; nr. 10-1149-01), as was serum and milk leptin (Biovendor, Modrice, Czech Republic; nr. RD291001200). Serum leptin, insulin, monocyte chemotactic protein-1 (mcp-1), interleukin-6 (IL-6), plasminogen activator inhibition (t-PAI), tumor necrosis factor alpha (TNF-α), and resistin concentrations were determined in the 21-day old offspring by the commercially available LINCOplex Mouse Adipokine Immunoassay kit from Linco Research (St. Charles, MO, US). All serum analysis was performed at the Laboratory for Health Protection Research, National Institute for Public Health and the Environment (Bilthoven, NL).

### Statistical analysis

All data are expressed as mean ± SEM. Statistical comparisons were performed with IBM SPSS Statistics version 20 (SPSS Inc., Chicago, IL, US). The effects of maternal diet on the body weight, calorific intake, serum profiles, arterial pressure, heart rate, and activity were analysed by Two-Way repeated measure ANOVA (diet × time), using the Greenhouse–Geisser correction for non-sphericity. NA data were analysed by Two-Way ANOVA considering maternal diet and sex as fixed factors. Variance in LF and HF were analysed using Model for Chart Software (AD Instruments, Colorado Springs, CO, US) and comparisons made using Student's *t*-test (two-tailed). Student's *t*-test (two-tailed) was used for all other analyses. Statistical significance was accepted at level of *P* < 0.05.

## Results

### Effect of sucrose feeding on maternal body weight, tissue mass, and milk and serum profiles

**Figure 1:**
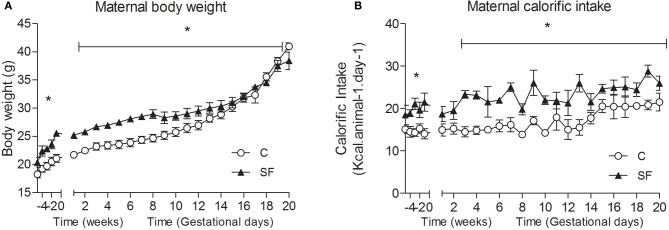
**(A)** Maternal body weight (g) and **(B)** calorific intake (kcal/animal /day) in dams fed either control (C, open symbols) or a sucrose rich diet (SF, closed symbols). ^*^P < 0.05 vs. control using two-way repeated measure ANOVA (diet × time) and Greenhouse–Geisser, *n* = 6–8 per group.

Body weight and calorific intake were significantly greater in sucrose fed dams (SF) compared with chow fed dams (C), during pre-pregnancy and throughout gestation (Figures 1A,B). At the end of pregnancy body weight converged, but at weaning SF dams were again heavier (body weight [g] SF, 27.5 ± 0.8 vs. C, 25.7 ± 0.2, *n* = 8, *P* < 0.05), and showed a significant increase in abdominal fat mass relative to body weight, compared with controls (maternal inguinal WAT weight [mg/g BW] SF, 15.5 ± 1.0 vs. C, 11.1 ± 1.6, *n* = 8, *P* < 0.05). At weaning, SF dams demonstrated a five-fold increase in fasting serum insulin, which was associated with a lower blood glucose concentration than control fed dams. No apparent difference in maternal serum leptin concentration was observed between groups at weaning (Table 1). There were no apparent differences in cholesterol or triglyceride concentrations between SF and control dams. The milk leptin concentration was significantly reduced in SF dams compared with controls (Leptin [ng/mL] SF, 1.2 ± 0.9 vs. C, 1.9 ± 0.2, *n* = 3, *P* < 0.05).

**Table 1:** **Maternal fasting serum cholesterol, triglyceride, glucose, insulin, and leptin concentrations at weaning in dams fed either control (*n* = 8) or a sucrose rich diet (*n* = 6)**.

**Group**	**Cholesterol (mmol/L)**	**Triglyceride (mmol/L)**	**Glucose (μmol/L)**	**Insulin (pmol/L)**	**Leptin (ng/ml)**
C	1.52 ± 0.11	0.81 ± 0.11	7.0 ± 0.4	88.1 ± 8.3	3.7 ± 0.6
SF	1.89 ± 0.17	0.97 ± 0.15	5.3 ± 0.4^*^	475.4 ± 14.5^†^	3.0 ± 0.4

* P < 0.05;

† P < 0.001 vs. offspring of control dams (two-tailed t-test). C indicates control dams; SF, sucrose fed dams. Values are mean ± SEM, n = 6–8 per group.

**Figure 2:**
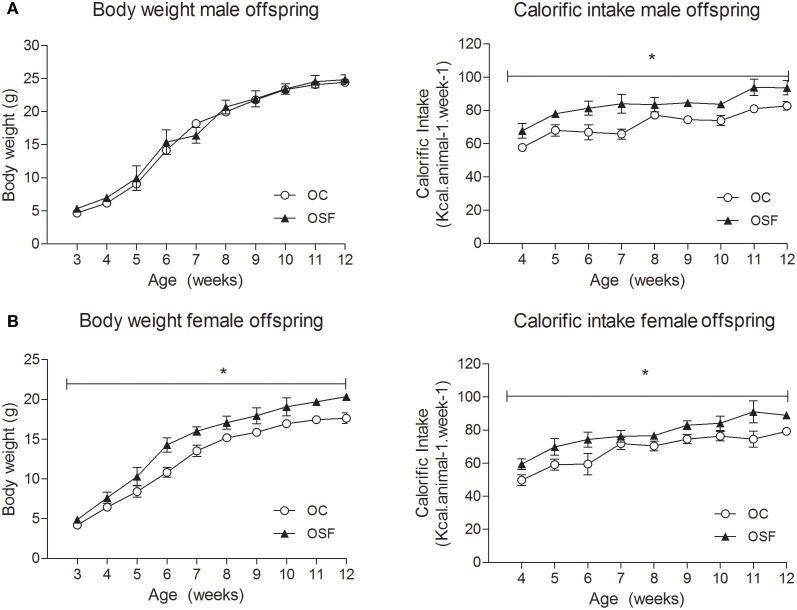
**Body weight (g) and calorific intake (kcal/animal/week) of (A) male and (B) female offspring of control (OC, open symbols) and sucrose fed dams (OSF, closed symbols).** ^*^P < 0.05 vs. control using two-way repeated measure ANOVA (diet × time) and Greenhouse–Geisser, *n* = 6–8 per group.

The maternal diet had no effect on litter size or pup survival rates. The newborn offspring of SF dams (OSF) were heavier than the offspring of chow fed dams (weight [g] OSF, 1.52 ± 0.14 vs. OC, 1.31 ± 0.04, *n* = 6, *P* < 0.01) but were of similar weight at weaning (3 weeks, Figures 2A,B). At weaning, female offspring of SF dams were hyperinsulinaemic (insulin [pmol/L] OSF, 48.8 ± 4.7 vs. OC, 28.3 ± 5.3, *n* = 6, *P* < 0.05) with increased serum IL-6 [pg/mL] OSF, 8.7 ± 1.9 vs. OC, 4.2 ± 0.5, *n* = 6, *P* < 0.01). Male OSF had increased plasminogen activator inhibitor concentrations (t-PAI [ng/mL] OSF, 2.3 ± 0.4 vs. OC, 1.5 ± 0.1, *P* < 0.05, *n* = 6). Serum resistin [ng/mL] was decreased in female offspring of SF dams (OSF, 3.0 ± 0.5 vs. OC, 4.6 ± 0.4, *n* = 6, *P* < 0.05). There was no apparent difference in the serum leptin concentrations between groups (leptin [ng/mL] OSF males 1.0 ± 0.2 vs. OC, 0.7 ± 0.1, *n* = 6, females, OSF 0.9 ± 0.1 vs. OC, 1.0 ± 0.2, *n* = 6).

### Effect of maternal sucrose feeding on offspring morphometry, serum profile, and glucose homeostasis

**Figure 3:**
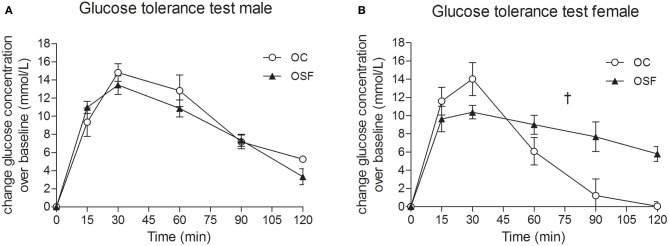
**Glucose tolerance tests at 3 months.** Blood glucose concentrations after administration of a bolus dose of glucose (1 g/kg, i.p.) in 3 month old male **(A)** and female **(B)** offspring of control (OC, open symbols) and sucrose fed dams (OSF, closed symbols). ^†^*P* < 0.01 vs. controls using two-way repeated measure ANOVA (diet × time) and Greenhouse–Geisser, *n* = 6–8 per group.

Calorific intake was greater in both male and female OSF vs. OC from 4 weeks of age, prior to a significant increase in body weight in the females at 6 weeks, but not in males (Figures 2A,B). At 3 months, female OSF, but not males, showed a significant increase in inguinal fat pad mass, heart weight, and liver weight, with reduction in mass of the *tibialis anterior* muscle and kidney (Table 2). This was accompanied by a 10-fold increase in the serum insulin concentration, a more than 2-fold increase in serum leptin, and an increase in triglyceride concentration in the female OSF (Table 3). The cholesterol concentration was not different from control in male or female OSF. At 3 months of age, glucose intolerance was evident in female OSF vs. OC, with a prolonged recovery phase in the glucose tolerance test (Figures 3A,B) and a greater area under the glucose concentration curve (glucose [mmol/L min] OSF, 1437 ± 124 vs. OC, 1077 ± 84, *P* < 0.05, *n* = 6). There were no apparent differences in the glucose response curves in male offspring.

**Table 2:** **Body Weight (BW), Inguinal fat pad mass, heart, liver, and muscle weights (tibialis anterior) of 3 months old offspring of control and high sucrose fed dams (*n* = 6–8 per group)**.

**Sex/group**	**Body weight (g)**	**Fat pad weight (mg/g BW)**	**Heart weight (mg/g BW)**	**Liver weight(mg/g BW)**	**Muscle weight (mg/g BW)**	**Kidney weight(mg/g BW)**
**MALE**						
OC	23.2 ± 0.4	12.7 ± 0.8	7.1 ± 0.3	38.9 ± 1.7	6.5 ± 0.2	17.4 ± 0.8
OSF	24.9 ± 0.7	12.4 ± 1.8	7.2 ± 0.4	39.7 ± 2.6	5.7 ± 0.5	16.9 ± 0.9
**FEMALE**						
OC	20.8 ± 0.4	11.7 ± 0.9	7.2 ± 0.2	37.4 ± 0.8	6.3 ± 0.2	15.4 ± 0.7
OSF	22.2 ± 0.1^*^	15.1 ± 0.7^*^	8.3 ± 0.3^*^	40.5 ± 1.0^*^	5.1 ± 0.2^†^	9.8 ± 0.6^$^

* P < 0.05;

† P < 0.01;

$ P < 0.001 vs. offspring of control dams (two-tailed t-test). C indicates offspring of control dams; SF, offspring of sucrose fed dams. Values are mean ± SEM, n = 6–8 per group.

**Table 3:** **Serum cholesterol, triglyceride, glucose, insulin, and leptin concentrations at 3 months old offspring of control (*n* = 8) or sucrose fed dams (*n* = 6)**.

**Sex/group**	**Cholesterol (mmol/L)**	**Triglyceride (mmol/L)**	**Glucose (umol/L)**	**Insulin (pmol/L)**	**Leptin (ng/ml)**
**MALE**					
OC	1.71 ± 0.27	0.37 ± 0.10	7.9 ± 0.9	39.3 ± 5.2	0.97 ± 0.02
OSF	1.66 ± 0.22	0.30 ± 0.06	7.9 ± 0.5	68.9 ± 10.2	1.20 ± 0.10
**FEMALE**					
OC	0.82 ± 0.12	0.34 ± 0.08	6.9 ± 0.8	20.3 ± 1.8	1.34 ± 0.26
OSF	1.23 ± 0.20	0.49 ± 0.11^*^	7.6 ± 0.8	200.3 ± 16.1^†^	3.82 ± 0.39^*^

* P < 0.05;

† P < 0.001 vs. offspring of control dams (two-tailed t-test). OC indicates offspring of control dams; OSF, offspring of sucrose fed dams. Values are mean ± SEM, n = 6–8 per group.

### Hemodynamic parameters in conscious offspring

**Figure 4:**
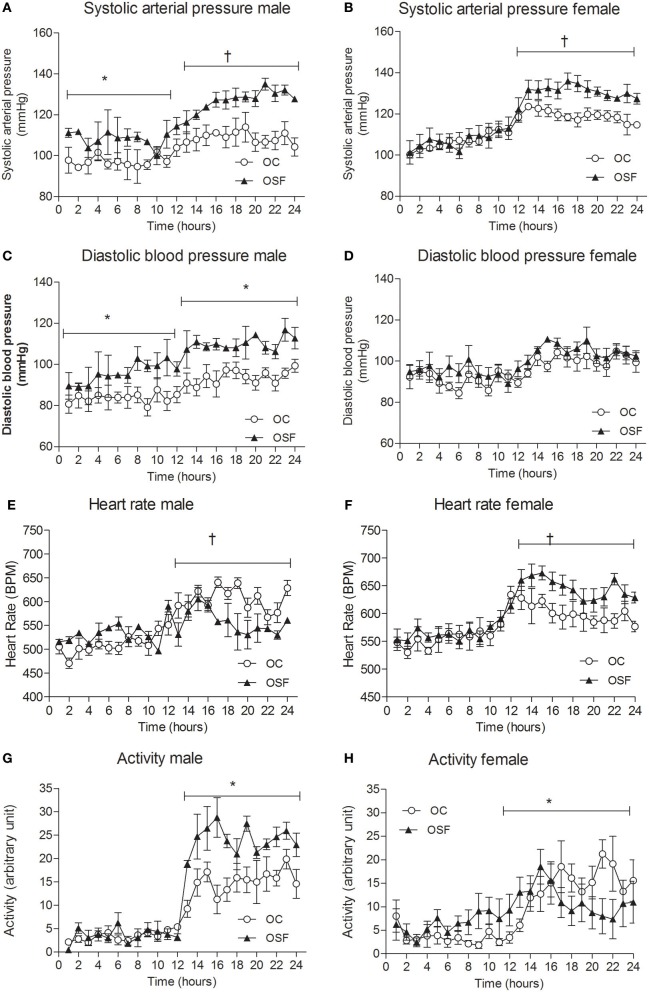
**Cardiovascular parameters at 3 months.** Systolic arterial pressure **(A,B)**, diastolic arterial pressure **(C,D)**, heart rate **(E,F)**, and locomotor activity **(G,H)** in 3-month old male and female offspring of control (OC, open symbols, *n* = 8) and sucrose fed dams (OSF, closed symbols, *n* = 6), ^*^P < 0.05; ^†^*P* < 0.01 vs. control, using two-way repeated measure ANOVA (diet × time) and Greenhouse–Geisser.

At 3 months, systolic arterial pressure (SAP) was significantly elevated in male OSF during day- and night-time and female OSF dams during active night phase only (Figures 4A,B, mean day-time SAP [mm Hg] male OSF, 113 ± 2 vs. OC, 97 ± 1, *P* < 0.05, mean night-time SAP [mm Hg] male OSF, 128 ± 1 vs. OC, 109 ± 1, *P* < 0.01; female SAP [mm Hg] OSF, 130 ± 1 vs. OC, 118 ± 1, *P* < 0.05, both day and night (Figures 4C,D, mean day-time DBP [mm Hg] male OSF, 95 ± 2 vs. OC, 84 ± 1, *P* < 0.05, mean night-time DBP [mm Hg] male OSF, 109 ± 1 vs. OC, 94 ± 1, *P* < 0.05, *n* = 6). Female night-time heart rate was increased in OSF vs. OC (HR [beats/min] OSF, 611 ± 10 vs. OC, 512 ± 11, *P* < 0.01, *n* = 6), whereas male offspring of OSF dams showed a reduction in heart rate compared with controls (HR [beats/min] OSF, 533 ± 6 vs. OC, 604 ± 5, *P* < 0.01, *n* = 6, Figures 4E,F). Locomotor activity during night-time was significantly increased in 3 months old male offspring of OSF dams vs. OC, whereas OSF females showed a reduction in locomotor activity compared with controls (Figures 4G,H). Spectral analysis of arterial pressure tracings showed increased low-frequency oscillations in OSF vs. OC (LF [nu] male OSF, 43 ± 3 vs. OC, 32 ± 5, *P* < 0.05, *n* = 6; female OSF, 52 ± 2 vs. OC, 36 ± 2, *P* < 0.05, *n* = 6). Low-frequency oscillations predominately reflect enhanced sympathetic activity. The high low frequency: high frequency ratio observed in OSF vs. OC (LF: HF [a.u], male OSF, 3.9 ± 0.2 vs. OC, 2.1 ± 0.3, *P* < 0.05, *n* = 6; female OSF, 4.3 ± 0.1 vs. OC, 2.5 ± 0.3, *P* < 0.05 is also indicative of a dominant increase in sympathetic activity.

### Tissue noradrenaline

**Figure 5:**
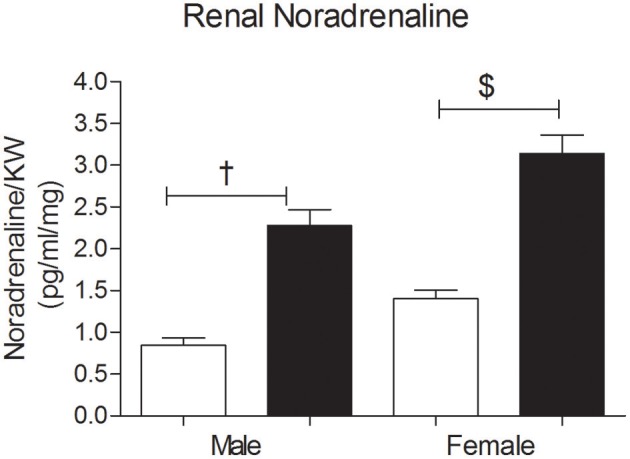
**Renal Noradrenaline (NA) concentrations (pg/ml per mg kidney weight) in 3 month old male and female offspring of control (OC, open symbols) and sucrose fed dams (OSF, closed symbols).** ^†^*P* < 0.01, ^$^*P* < 0.001 vs. control, two-way ANOVA (diet × sex), *n* = 6–8 per group, KW, kidney weight.

The renal NA concentration was significantly increased in the OSF male and female offspring compared with controls, at 3 months of age (Figure 5). ANOVA showed an association between sex and renal NA concentration, with increased NA concentration in females.

## Discussion

This study has shown persistent and deleterious effects of a sucrose-rich diet in pregnancy on offspring cardiovascular and metabolic function and contributes to the growing evidence that maternal nutritional imbalance can adversely influence the mechanisms underlying development of normal cardiovascular and metabolic function. Importantly, the data suggest that a maternal diet rich in free sugars, comparable to that commonly consumed in the UK (approx. 21% of total energy) and worldwide (Whitton et al., 2011), can have far reaching consequences for offspring health.

A sugar-rich diet *in utero* and during the suckling period led to profound hyperinsulinaemia, increased adiposity and impaired glucose tolerance in the female offspring. A relevant study by Srinivasan et al. (2006) has shown a similar adult phenotype in neonatal rats artificially reared via intragastric cannulas on high-carbohydrate (HC) formula milk (56% of the calories from carbohydrate, compared to typically 8% in rat milk). Female HC offspring developed chronic hyperinsulinaemia and adult-onset obesity at 12 weeks of age, highlighting the potential importance of the suckling period, albeit with this artificial feeding regimen. In contrast, the present study which investigated the effect of a maternal high sucrose diet during both prepregnancy through to suckling is arguably more relevant to the human situation. Recently, similar comparison was made in rats (Sedova et al., 2007). A high sucrose diet (70% calories as sucrose) was provided to inbred PD/Cub rats, which display a metabolic syndrome like phenotype, one week before breeding, and throughout pregnancy and suckling (Sedova et al., 2007). Contrary to our observations, this study revealed no increase in maternal weight gain or food intake in SF dams compared with control dams. This might be explained by the palatability of the different sucrose diets or the difference in genotype. On the other hand, both studies demonstrated increased birthweight in offspring of the SF dams, which is likely to be attributable to the reduced maternal glucose tolerance and subsequent fetal hyperinsulinaemia (Sedova et al., 2007).

Both male and female offspring of the SF dams showed enhanced appetite (hyperphagia) which was associated with increased body weight and greater inguinal fat mass in female offspring at 9 weeks of age. An insight into a potential mechanism is provided by the early studies of Plagemann and colleagues (Schmidt et al., 2000) which showed that overfeeding in the postnatal period by small litter rearing leads to persistent alteration of hypothalamic appetite control. Hyperinsulinaemia, as well as an increase in intrahypothalamic insulin concentrations was suggestive of resistance to insulin in the hypothalamic appetite regulatory centers, in which reduction in the anorexic effects of insulin would fail to suppress food intake leading to hyperphagia (Plagemann, 2008). A similar centrally mediated mechanism may occur in the offspring of the SF dams since the female offspring demonstrated a marked increase in plasma insulin concentration. Maternal plasma insulin profiles at weaning were reflected in the hyperinsulinaemic status of the weanling female offspring, with no apparent change in maternal plasma leptin. This therefore implicates the 5-fold increase in insulin in the SF dams and/or transient hyperglycaemia in the non-fasted insulin resistant state as potential modulators of hypothalamic function in this model. The maternal hyperinsulinaemia in this and other sucrose feeding studies is likely to be the consequence, not of increased fat mass, which was only very modest in the dams, but because of the well recognized hepatic enzymatic response to fructose (a component of sucrose) which leads to increased glucose outflow and raised pancreatic insulin secretion (Le and Tappy, 2006). In these fasted animals, maternal hyperinsulinaemia was not associated with hyperglycaemia, rather a reactive hypoglycemia, which is likely to reflect the “rebound” from the dietary induced hyperinsulinaemia (Sacca et al., 1979).

Mechanistically, insulin has been implicated in the developmental dysregulation of specific appetite regulatory *neuropeptidergic* neurons in the arcuate nucleus (ARC) with reported increased activity of the orexogenic peptides galanine and neuropeptide Y (NPY), thus predisposing to an obese phenotype characterized by hyperphagia (Plagemann et al., 1998, 1999). Perinatal hyperinsulinaemia in the immature hypothalamus can also cause permanent morphological changes of the ventromedial hypothalamus nucleus (VMN) and the ARC, regulating metabolism and body weight (Dorner et al., 1988; Dorner and Plagemann, 1994; Plagemann et al., 1999). Future neuroanatomical studies using immunohistochemical methods will determine the extent of neuronal maldevelopment in this model and address the intriguing possibility that altered methylation status of relevant genes e.g., anorexigenic peptide proopiomelanocortin (POMC), Insulin receptor, may play a permissive role (Minth-Worby, 1994; Plagemann et al., 2010).

The maternal and neonatal leptin profile has also been strongly implicated in “hardwiring” the appetite regulatory system (Bouret et al., 2008; Kirk et al., 2009). However, without any evidence for hyperleptinaemia in dam, milk, or weanling animals, a predominant role for hyperinsulinaemia in this model is suggested. However, the low concentration of leptin in the milk of the SF dams might have led to suboptimal development of hypothalamic neurons involved in energy balance (Bouret et al., 2004) and predispose to offspring obesity (Stocker et al., 2007; Palou and Pico, 2009). Recent studies suggest a protective effect of neonatal leptin supplementation against subsequent diet-induced obesity (Pico et al., 2007; Sanchez et al., 2008). Oral supplementation of physiological concentrations leptin in rat pups throughout lactation resulted in decreased food intake, altered food preference, and improved leptin sensitivity in adulthood (Pico et al., 2007). However, others have demonstrated that exogenous leptin administration in mice (2.5 μg/g s.c.) during the early postnatal period (PD5-10) led to accelerated weight gain on a high fat diet compared to saline treated controls (Yura et al., 2005). Similar increases in susceptibility to diet induced obesity have been reported in neonatal rat pups administered leptin at a similar dose (PD3-13) (Vickers et al., 2008). It would appear that the effects of early exposure to leptin on the developmental programming of energy balance in rats and mice may be critically dependent on the timing, dose, duration, and route of administration of leptin. Sugar metabolism also interacts with motivational and reward regulated by the dopamine- and μ-opioid receptor system (Figlewicz et al., 2007). Early exposure to sucrose could therefore alter the adult motivation behavior and may constitute an important factor determining adult feeding behavior and energy expenditure.

Despite hyperphagia in both male and female offspring of SF dams, only females exhibited an increased body weight, compared with controls, which might reflect their reduced locomotor activity. Interestingly, a previous study has implicated AgRP neuronal expression of STAT3, a signaling molecule in the insulin and leptin pathway, in the regulation of locomotor activity in mice (Mesaros et al., 2008). Hence reduced STAT3 signaling secondary to insulin resistance in the offspring of SF dams might be expected to reduce activity in the obese/hyperleptinaemic females, and shall be further explored. Inguinal fat mass, and liver weight were increased in females whilst muscle mass was decreased. Both increased adiposity and reduced muscle mass would likely contribute to the impaired glucose tolerance observed. Decreased muscle mass impairs the utilization of glucose, which reduces the insulin sensitivity (Krentz, 1996) and visceral adiposity is considered to be an important determinant of insulin resistance and secretion (Wagenknecht et al., 2003). The increase in serum triglycerides in female offspring of SF dams also would support the free fatty acid theory of insulin resistance (Ginsberg et al., 2005). A novel observation amongst models of fetal overnutrition is that the female offspring showed a raised serum IL-6 concentration as young animals compared to controls, and a reduction in resistin, implicating these adipokines in development of insulin resistance (Hotamisligil et al., 1995).

Male and female offspring of the SF mice showed elevated SAP compared with controls whereas females offspring alone showed an increase in heart rate. The increase in heart rate may reflect a failure of the normal baroreceptor response to increasing arterial pressure and implicates altered autonomic control. The decrease in insulin sensitivity in the female offspring might contribute to the baroreflex impairment. Brain insulin has been shown to improve baroreflex function in both humans (Young et al., 2010) and animals (Okada and Bunag, 1994; Pricher et al., 2008).

We hypothesize that sympathetic over-activity may play an important role since sympathetic component (low frequency and high: low frequency ratio) of HRV was elevated in both male and female offspring of SF dams. Renal NA was also raised which could also reflect increased sympathetic tone. The marked hyperinsulinaemia, in female offspring, acting through centrally mediated effect via the hypothalamus may contribute to altered sympathetic activity and baroreflex activity leading to hypertension (Muntzel et al., 1995; Chapman and Sposito, 2008). Similarly the hyperleptinaemia in female offspring may activate renal sympathetic nerve activity, which subsequently increases the renin angiotensin system and decreases sodium excretion, contributing to hypertension (Rahmouni et al., 2005). This cannot explain the hypertension in the males, however, as they were not fatter than controls. Altered renal sympathetic drive and central sympathetic outflow has previously been implicated in the etiology of the hypertension arising from maternal undernutrition, in the spontaneously hypertensive rat (Perez et al., 2006; Da Silva et al., 2008), high fat feeding rabbits (Armitage et al., 2012) and in our report in offspring of diet induced obese dams which showed increased arterial pressure and sympathoexcitatory activation prior to the onset of obesity (Samuelsson et al., 2010). In a recent study in which we attempted to isolate the consequences of the fat component of the obesogenic diet on offspring arterial pressure the data suggested that maternal obesity rather than fat diet per se is an important determinant of hypertension for both males and females (White et al., 2009; Rudyk et al., 2011). This also has parallels in the human literature where greater maternal prepregnancy weight (Fraser et al., 2010; Hochner et al., 2012) and maternal weight gain in pregnancy (Fraser et al., 2010; Mamun et al., 2010; Hochner et al., 2012) are associated with adverse cardiovascular risk in the children.

The evaluation of known cardiovascular risk factors in the young animals showed an in increase in the serum plasminogen activator inhibitor −1 (PAI-I) (Agirbasli, 2005; Bieswal et al., 2006; Jensen et al., 2008), further accentuating the early origins of cardiovascular risk in this model. The increased heart weight in the female offspring may be a primary event or secondary to hypertension.

We had initially hypothesized that the sucrose-rich diet would lead to a similar offspring phenotype previously reported in the offspring of obese mice fed a highly palatable diet (Samuelsson et al., 2008). This hypothesis was not proven, as we observed no metabolic changes in male offspring of the SF dams, in which the metabolic phenotype was predominant in the previous study (Samuelsson et al., 2008). The only commonality was the hypertension, observed in both male and female offspring in both models. The differences may be explained by the maternal phenotype since the SF dams showed only a 2-fold increase in abdominal fat mass, compared with a 4-fold increases in the dams fed the obesogenic diet (Samuelsson et al., 2008). Maternal insulin was also markedly different being significantly elevated in the SF animals. We cannot exclude the contribution of the increased fat content of the maternal “obesogenic” diet in the previous study (16% fat vs. 5% fat w/w). The protein content of 12% w/w in both studies is unlikely to have played a role in either protocol (Langley and Jackson, 1994).

Several animal models of developmental programming exhibit sex differences in the response to a prenatal insult, with the male and female phenotype being dependent on the timing and severity of the insult (Gilbert and Nijland, 2008). The current findings indicated that female offspring were more susceptible to excess maternal intake of dietary sucrose, having increased adiposity and greater disturbance of glucose homeostasis. Thus, male sex hormones may be protective against the prenatal exposure or female hormones may further compromise disturbances in pathways of lipid and glucose metabolism. Further studies in which males are castrated or females ovariectomized in early life are needed to investigate these possibilities.

In conclusion, a sucrose-rich diet in pregnant mice leads to exacerbation of factors associated with cardiovascular risk in the adult offspring, which were most evident in the female offspring. Relationships between sucrose consumption in women and similar parameters of disease risk in their children should be explored.

### Conflict of interest statement

The authors declare that the research was conducted in the absence of any commercial or financial relationships that could be construed as a potential conflict of interest.
